# Notes on Liquid Articles of Dietary

**Published:** 1906-09-15

**Authors:** 


### Progress in Medicine and Surgery.

### NOTES ON LIQUID ARTICLES OF DIETARY.

**Milk and its Preservatives.**—The addition of preservatives to milk is far from being an uncommon practice, and in general is looked upon with disfavour both by the public and by sanitarians. Boric acid and salicylic acid are frequently employed, but recently formic aldehyde in the form of its watery solution, formalin or formol, has come into use, and the extremely small quantities which will suffice to prevent "souring" make it especially suitable for the purposes of the milk vendor. Although, on the whole, medical opinion is against the use of formalin, some medical writers of standing have urged that, under proper restrictions, milk preservation by this means might with advantage be legalised.[1] Their argument is briefly as follows: Much milk is wasted because after, say, 24 hours, it turns sour and becomes obviously unfit for food. Formalin, in the proportion of 1 in 20,000 to 1 in 30,000, considerably delays this souring, enabling the milk to be kept and used for quite 48 hours. The precise action of formalin is to inhibit the growth of the bacillus acidi lactici, and it may also have an inhibitory effect on the other bacteria in milk which produce less obvious, but no less deleterious, changes. In these small amounts the taste of the milk is not affected, and it cannot be shown experimentally that milk curdling or milk digestion are affected. Moreover, there are a few observations on the human subject which show that no ill effects follow the ingestion of milk thus preserved. The last observations are, however, far too few to justify general conclusions, and before the use of formic aldehyde even in 1 in 30,000 of milk, is legalised, much more extended experience of its effect on the body processes will be necessary. Meanwhile, the advocates of formalin-milk advise that all milk so treated should have the amount of preservative clearly stated thereon, but we doubt whether, considering that milk is not sold done up in packets, any such notification of the added substance would reach the majority of the consumers.

*Tests for Formalin.*—Any medical man can easily satisfy himself of the presence or absence of formalin in milk, as several very reliable and delicate tests exist. (1) Hehver's test. Take 5 c.c. of 94-per-cent. *commercial* $H_2SO_4$ (which contains traces of perchloride of iron) in a test-tube and gently pour on to it milk diluted with an equal volume of water. If formalin is present a violet ring forms at the junction of the fluids. (2) Jorissin's test: 10 c.c. of milk is placed in a test-tube and 1 c.c. of 1-per-cent. aqueous solution of phloroglucin and 3 c.c. of 10-per-cent. solution of caustic potash are added. A pink, light pink, or brown colour will develop in a few seconds if formic aldehyde is present.

**Formalin** postpones the bacterial changes in impure milk, and thus treated milk at the end of 48 hours is, from the bacteriological point of view, the same as untreated milk at the end of 24 hours. A process recently described[2] as the Budde system apparently does more than this, as it actually destroys the organisms present without the application of undue heat, which all physiologists are now agreed is prejudicial to the quality of this food. The milk is warmed (118°—122° F.) with .35 per cent. peroxide of hydrogen. At this temperature it is kept with constant stirring for half an hour, and is then heated to 125° F. for two or three hours. It seems clearly shown that the action of the $H_2O_2$ is powerfully germicidal, and that milk so treated keeps well for more than a week. It is also claimed that milk digestion is not interfered with, the clotting by rennet being only delayed and a more flocculent coagulum resulting. Whether milk so treated is liable to have the same unfavourable effect in cases of infantile scurvy as ordinarily Pasteurised milk has sometimes been observed to produce, is not at present known. It seems, however, quite clear that the peroxide produces a much more marked effect on the bacteria in milk than mere Pasteurisation.

**Tea and its Abuse.**—The abuse of tea, if not so widely spread as the abuse of alcohol, is, at any rate, sufficiently important to occupy the attention of the medical profession. The word abuse implies that, as beverages, both these substances have a corresponding use, and it is highly probable that primarily the objects which induced men to employ them were identical in both cases.[3] In primitive, or even in certain civilised states, the use of water, usually obtained from shallow wells, is not without serious dangers. The fact that alcoholic drinks are practically sterile, and that to make tea the water must be boiled, suggests that one of the objects attained by their use is the avoidance of microbic diseases. This, for instance, is in all probability the main reason for the large consumption of tea in Russia, where it is drunk in so weak an infusion that its physiological effect on the organism must be, to all intents and purposes, non existent. The same may be said of the weak wines drunk so largely in many vine-growing countries.

**Stimulation** is the direct physiological action. The word, strictly speaking, is something of a misnomer. The action of alcohol and of caffeine on the nervous system is primarily narcotic. By slightly deadening sensation they remove not only certain visceral sensations, such as hunger, but the less definite cerebral perceptions of worry, care, fatigue, and so enable men to draw on their reserve of energy and go straight for any object on which they wish to concentrate their powers. This, no doubt, is the main function of these beverages in our ordinary life of to-day, and many will be found to maintain that this function is a perfectly legitimate one provided it is not carried to excess. Persistent slight "stimulation" of this sort often, of course, in the long run lead to disastrous results; but in this respect tea or alcohol are not more likely to lead to neurasthenia or nervous breakdown than persistent voluntary over-exertion of the mind or body on the part of a rigid abstainer.

The effects of the abuse of tea are, perhaps, not widely realised. They arise from two sources. Firstly, "strong" tea, owing to the high proportion of tannin which it contains, is a powerful gastric irritant. Moreover, if taken with food, a similar effect is produced on proteid articles of diet as is observed on the gastric mucous membrane. Meat is tanned and rendered hard and indigestible, hence "meat-teas" are to be condemned. Bacon, on the other hand, having already been treated in the curing process, is practically unaffected by tannin. The second effect is on the nervous system. It does not seem certain that the tremors and other nervous manifestations seen in excessive tea-drinkers, or in professional tea-tasters, are solely due to the caffeine. Green tea, for instance, which produces much more severe nervous symptoms than black tea, contains no higher proportion of caffeine. But in any case the serious damage to the nervous system produced by excessive tea bibbing cannot be overlooked, and may, in the opinion of some good authorities, actually culminate in insanity.

[1] Scot. Med. and Surg. Jour., Nov., 1905, p. 398. [2] Pract., Jan., 1906, p. 132. [3] Pract., Jan., 1906, p. 38.

